# Atypical Parathyroid Tumor Causing Primary Hyperparathyroidism With a Clinical Presentation of a Brown Tumor

**DOI:** 10.1210/jcemcr/luaf154

**Published:** 2025-07-11

**Authors:** Nathanaël Saison, Sandra Fatio, Dominik Pytlik, Stephan Rauthe, Roger Schmid, Chantal Stutz

**Affiliations:** Department of Surgery, Hospital Center Biel/Bienne, Biel/Bienne 2502, Switzerland; Endocrinology and Diabetes, Medical Center EndoDia-Centre, Biel/Bienne 2502, Switzerland; Endocrinology and Diabetes, Medical Center EndoDia-Centre, Biel/Bienne 2502, Switzerland; Institute of Pathology, Viollier AG, Allschwil 4123, Switzerland; Department of Surgery, Hospital Center Biel/Bienne, Biel/Bienne 2502, Switzerland; Department of Surgery, Hospital Center Biel/Bienne, Biel/Bienne 2502, Switzerland

**Keywords:** parathyroid, brown tumor, atypical parathyroid tumor

## Abstract

Brown tumors are rare osteolytic bone lesions linked to primary hyperparathyroidism (pHPT), caused by excessive parathyroid hormone (PTH) production. They feature microfractures, hemorrhage, and hemosiderin deposition. Atypical parathyroid tumors (APT) are uncommon parathyroid neoplasms with histologic features resembling carcinoma but without definitive parathyroid carcinoma (PC) criteria. APTs can rarely lead to brown tumors. We report a 53-year-old male with a history of nephrectomy for renal cell carcinoma and Hodgkin disease. Imaging revealed a growing osteolytic lesion in the left iliac bone. Laboratory findings indicated pHPT with elevated PTH levels. Sonography and ^18^F-fluorocholine positron emission tomography–computed tomography (PET-CT) identified a right inferior parathyroid tumor. The patient underwent focused parathyroidectomy, and histology confirmed APT with fibrous tracts and trabecular growth features. APT accounts for 0.5% to 4.4% of parathyroid surgeries and may rarely lead to brown tumors. Differentiating brown tumors from malignancies requires biochemical, imaging, and histopathological evaluation. Although APTs generally follow a benign course, long-term surveillance is essential, particularly in familial cases. Further research is needed to investigate the malignancy potential and recurrence risk of rare atypical parathyroid neoplasms. This case highlights the importance of a multidisciplinary approach in their diagnosis and management.

## Introduction

Brown tumors are rare osteolytic bone lesions associated with primary hyperparathyroidism (pHPT). They occur due to excessive parathyroid hormone (PTH) production, which stimulates osteoclastic activity and bone resorption, leading to demineralization and cystic changes in the bone, resulting in microfractures, hemorrhage, hemosiderin deposition, and excessive vascular proliferation, giving rise to the term brown tumor [1]. These lesions can be challenging to differentiate from malignancies based on imaging alone, necessitating biochemical evaluation, including PTH, calcium, and phosphate levels, for accurate diagnosis [2]. The rarity of atypical parathyroid tumors (APT) complicates understanding their role in conditions like brown tumors. APTs represent neoplasms with histological features suggesting malignancy but lack definitive criteria for parathyroid carcinoma (PC) [3]. Brown tumors related to APT are exceedingly uncommon, but cases highlight their potential to manifest in hyperparathyroidism, underscoring the need for a multidisciplinary approach for proper diagnosis and management. We present a rare case of a patient exhibiting a brown tumor as a clinical manifestation of APT.

## Case Presentation

**Figure 1.**
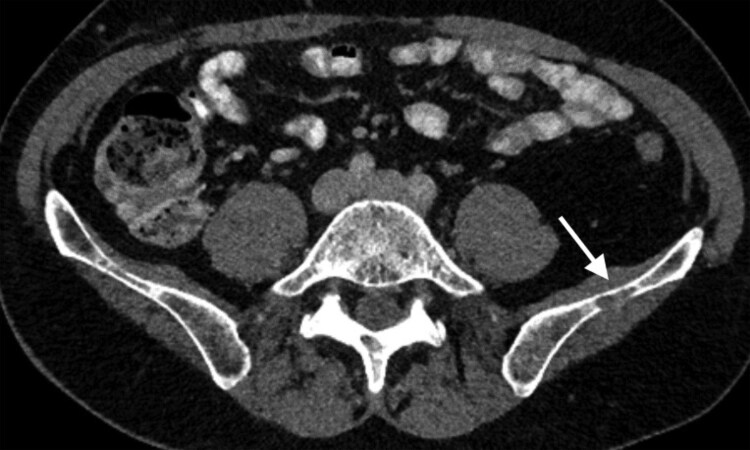
CT scan imaging demonstrates a 5 × 2 mm osteolytic lesion in the left iliac bone with cortical destruction (white arrow).

A 53-year-old male individual was referred from our urology unit 6 months after undergoing a nephrectomy for 2 renal cell carcinomas (pT1b [m], G3, Intermediate Risk per Leibovich). His medical history included Hodgkin disease, in complete remission for 3 decades following chemotherapy and stem cell transplantation. A computed tomography (CT) scan revealed a newly developed osteolytic lesion in the left iliac bone, measuring 5 × 2 mm, with cortical destruction. Follow-up CT imaging 3 months later showed the lesion had grown to 18 × 4 mm (Fig. 1).

## Diagnostic Assessment

**Figure 2.**
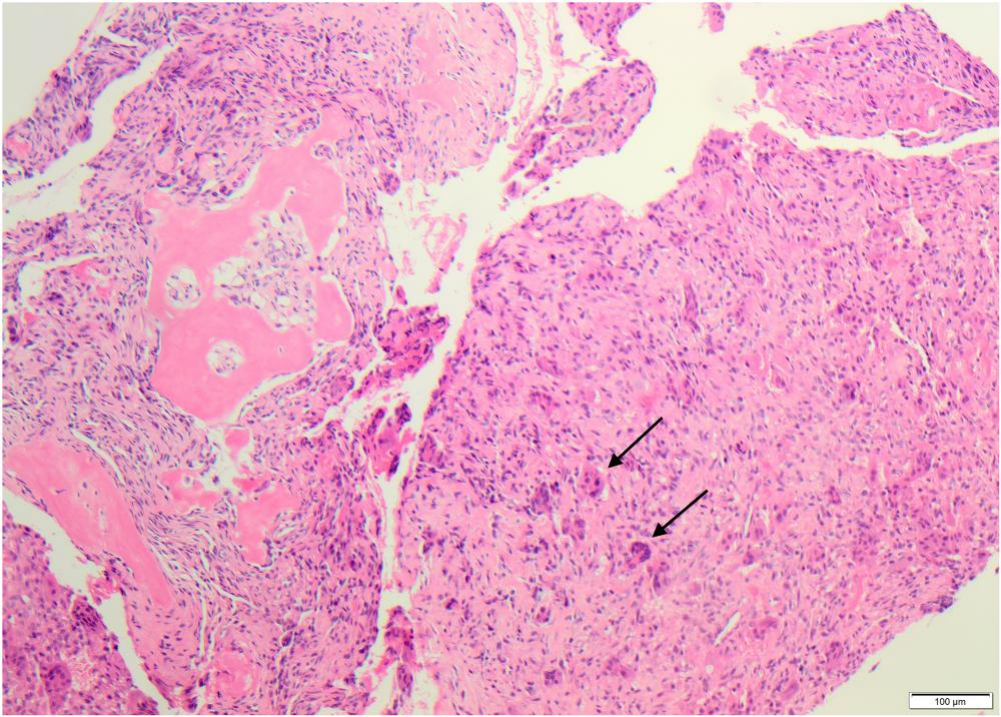
Histological examination of the bone biopsy reveals fibroblastic tissue with scattered multinucleated osteoclastic giant cells (indicated by arrows), consistent with features of a brown tumor (hematoxylin and eosin; 100×).

**Figure 3.**
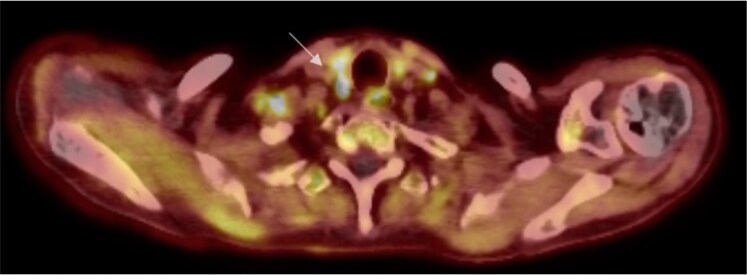
An ¹8F-fluorocholine PET/CT scan revealed a choline-avid lesion located dorsally to the right of the thyroid, consistent with a parathyroid tumor (white arrow).

Pathological examination of the iliac bone lesion demonstrated monomorphic spindle cell proliferation with numerous osteoclast-like giant cells and a gray-brown fragment of hard bone (Fig. 2). Laboratory findings confirmed primary hyperparathyroidism, with elevated PTH levels of 187 pg/mL (SI: 187 ng/L) (reference range, 15-68.3 pg/mL [SI: 15-68.3 ng/L]) despite normal serum calcium levels. Sonography and ^18^F-fluorocholine positron emission tomography–computed tomography (PET-CT) localized a right caudal parathyroid tumor (Fig. 3). The clinical, radiological, biochemical, and histological findings led to a diagnosis of a brown tumor. A focused parathyroidectomy was indicated.

## Treatment

**Figure 4.**
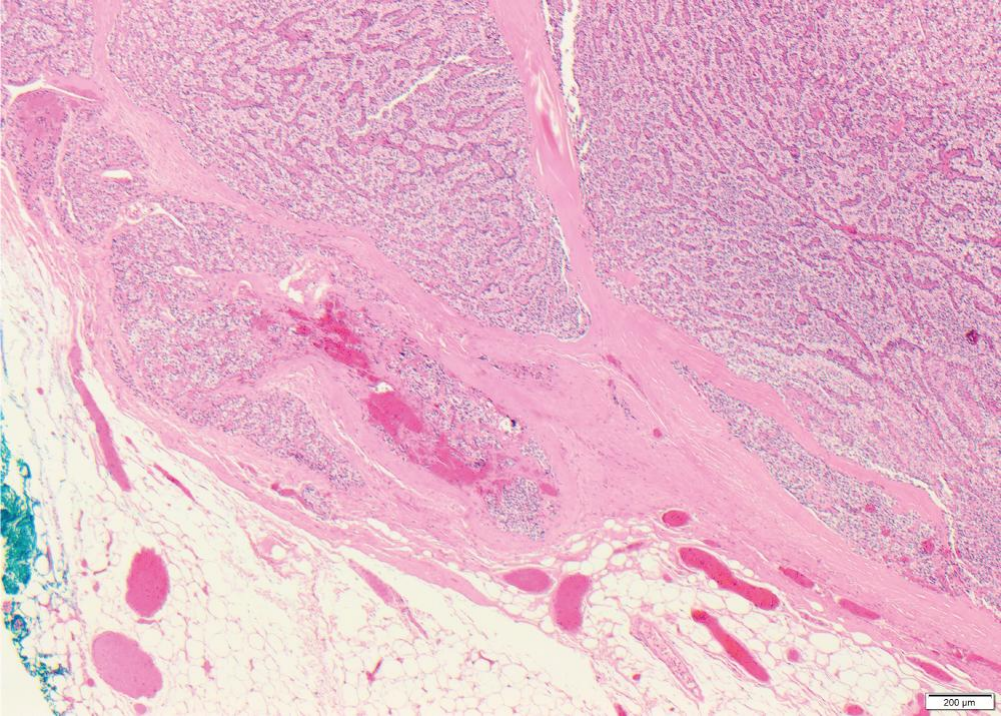
Histological features of atypical parathyroid tumor with trabecular, nested, and solid areas, and irregular fibrous bands (hematoxylin and eosin; 40×).

The patient underwent a focused parathyroidectomy. Intraoperatively, a dark yellow, shimmering structure approximately 3 mm in size was identified laterocaudally to the right lower pole of the thyroid gland. Histological examination confirmed parathyroid tissue exhibiting partial septation due to broad fibrous tracts or areas of embedded fibrosed soft tissue, with a trabecular and solid growth pattern. No evidence of atypical features or increased mitotic activity was observed. Immunohistochemical analysis for Ki-67 demonstrated a heterogeneous distribution of positively stained cells, with a maximum count of 5 per 100 cells. There was no indication of necrosis, nor any signs of infiltration into blood vessels, lymphatic vessels, or perineural spaces (Fig. 4).

## Outcome and Follow-Up

The patient demonstrates normal postoperative regression and normalization of primary hyperparathyroidism. Postoperative PTH levels dropped to 26 pg/mL (SI: 26 ng/L), corresponding to 19.84% of the last preoperative value. The patient has no symptoms related to postoperative hyperparathyroidism and no significant complications following the surgery. Follow-up CT imaging at 6 months after parathyroidectomy shows stable disease with a slight reduction in the size of the brown tumor.

## Discussion

APT—previously referred to as atypical parathyroid adenoma—has been reclassified since the World Health Organization (WHO) Update in 2022 [4]. APT is a rare entity, with an incidence ranging from 0.5% to 4.4% among individuals undergoing parathyroid surgery [5]. APTs are usually sporadic but may also occur in a familial setting, particularly in conditions such as multiple endocrine neoplasia (MEN) syndromes or hyperparathyroidism-jaw tumor syndrome (HPT-JT) [6]. These tumors are considered neoplasms of uncertain malignant potential, exhibiting worrisome histologic features that may resemble carcinoma. Features such as intratumoral fibrous bands, cytologic atypia, occasional mitoses, necrosis, and hemorrhage may be present [7]. However, definitive malignant features like capsular invasion, vascular invasion, or metastasis are absent, emphasizing the importance of meticulous pathological examination to distinguish APT from PC. The current WHO criteria may contribute to misdiagnosis, as both aldosterone-producing adenomas (APAs) and PCs share overlapping histological features, such as solid growth patterns and cellular atypia. Additionally, relying solely on clear signs of local invasion or metastasis may result in the underdiagnosis of early-stage carcinomas or those with subtle malignant features. APTs are considered intermediate entities between parathyroid adenomas and parathyroid carcinomas; they may represent a precursor to invasion and have the potential to progress to PC. However, the exact risk and mechanisms of malignant transformation remain unclear, and further research is needed to establish a definitive link between APTs and the development of PC. Careful pathological evaluation and close clinical follow-up are recommended to monitor for any signs of progression [8]. Histochemical markers, particularly the loss of parafibromin staining, have been associated with APT and may indicate a risk of malignant transformation, as observed in some cases of PC [9]. The biochemical profile of patients with APT typically resembles that of PC. It is often characterized by normal calcemia or moderate hypercalcemia associated with elevated levels of PTH, leading to pHPT [10]. Recurrence and relapse of APT occur in approximately 3% of cases, with familial cases, such as those associated with multiple endocrine neoplasia (MEN) syndromes or hyperparathyroidism-jaw tumor syndrome (HPT-JT), exhibiting a higher recurrence rate compared to sporadic cases. Variant of the *CDC73* tumor suppressor gene, which encodes parafibromin, is known to be associated with HPT-JT and has also been found frequently in sporadic PC. Less frequently, *CDC73* variants have also been observed, being most common anomaly in APT. Furthermore, a study shows that, at the time of follow-up, 42% of APT patients carrying germline *CDC73* variants exhibited recurrence and persistence of the disease [5]. Recurrence risk, although low (∼3%), is increased in familial cases, particularly those with *CDC73* variants. These patients may require more aggressive surveillance strategies, including genetic testing and frequent follow-up assessments.

Continuous excess production of PTH disrupts calcium-phosphate metabolism and induces osteoclast activation. This, along with the local production of growth factors and cytokines, leads to osteolytic changes characterized by increased bone turnover and fibrous tissue replacement. Elevated calcium levels can cause vascular damage, resulting in hemorrhage and hemosiderin deposition, which contribute to the characteristic brown pigmentation seen in brown tumors [11]. These pathological changes most commonly occur in the pelvis, ribs, clavicle, mandible, femur, and other long bones. Brown tumors are rare, affecting less than 2% of patients with hyperparathyroidism, and typically arise due to prolonged or severe pHPT. Although this mechanism has little to do with the histopathology of APTs, clinical manifestations in this context remain rare. The radiological features of brown tumors can be striking and are often identified as incidental findings on imaging studies such as CT or magnetic resonance imaging (MRI) scans. These lesions may exhibit characteristics that mimic malignancy, including irregular bone destruction and cortical thinning, leading to potential diagnostic confusion [12]. This highlights the importance of a comprehensive diagnostic approach that includes biochemical analysis of calcium and PTH levels, imaging studies, and histopathological examination of the lesions. To avoid misdiagnoses and ensure accurate identification, a multidisciplinary approach is essential, involving close communication among endocrinologists, radiologists, pathologists, and surgeons. Histological confirmation is critical to distinguishing brown tumors associated with APT from malignant bone lesions or other neoplastic processes. For certain lesions, parathyroidectomy alone is sufficient to treat the condition. Surgical intervention often leads to regression of the associated brown tumors and normalization of osteoclastic activity, correcting the underlying imbalance in bone remodeling. Postoperative improvements in bone density and resolution of skeletal symptoms have been reported in many cases [13]. The non-treatment of hyperparathyroidism leads to persistent pain, musculoskeletal weakness, bone erosion, bowing of the bones, and fractures. Regarding surveillance, regular monitoring of calcium levels, PTH levels, and bone density, along with periodic imaging studies, is essential to assess disease progression and prevent complications.

Regarding surveillance and follow-up, current practices vary widely and are often institution-specific, reflecting differences in expertise and available resources. Literature suggests that approximately 90% of brown tumors undergo near-complete resorption within 6 to 10 months after parathyroidectomy [14]. However, in some cases, resorption may take significantly longer (2-5 years), particularly in patients over 60 years of age or those with very large tumors. It is essential to rule out metabolic causes of impaired bone mineralization in cases of delayed regression [15]. Interestingly, although rare, there have been reports of paradoxical tumor progression after parathyroidectomy, emphasizing the importance of close postoperative monitoring and follow-up imaging to assess bone remodeling dynamics. Paradoxical progression can occur, for example, when bone remodeling is delayed due to the sudden drop in PTH levels after surgery, or in the case of a transient increase in bone resorption as part of hungry bone syndrome. Recommendations generally emphasize regular monitoring of calcium and PTH levels, periodic imaging studies, and clinical assessments to detect any signs of disease recurrence or progression. Emerging evidence suggests that incorporating genetic testing and molecular profiling in familial cases may provide additional insights into the risk stratification and management of APT.

This case presents a rare clinical manifestation of hyperparathyroidism with a brown tumor, resulting from an APT. The brown tumor was incidentally identified, and its radiological and histopathological features closely mimicked a giant cell tumor. Accurate differentiation was achieved through laboratory tests, particularly PTH and calcium assays, which are essential to avoid misdiagnosis. Although APTs are uncommon, they can present with malignancy-suggestive histological features, making careful pathological examination critical for distinguishing them from PC. Despite their resemblance to malignant tumors, APTs generally follow a benign course, with recurrence rates remaining low. Surgical treatment, such as focused parathyroidectomy, effectively resolves symptoms and prevents complications like brown tumors. Ongoing surveillance is especially important in familial cases, where recurrence rates are higher. Due to the rarity of these tumors, further research and a multidisciplinary approach are necessary to enhance diagnostic accuracy, optimize treatment strategies, and improve long-term management.

## Learning Points

- Brown tumors are rare osteolytic bone lesions associated with primary pHPT and they can mimic malignancies on imaging. Their occurrence in association with APT is exceptionally rare, highlighting the need for thorough biochemical and histopathological evaluation to avoid misdiagnosis.
- Collaboration between endocrinologists, radiologists, pathologists, and surgeons is essential for accurate diagnosis and optimal management.
- Focused parathyroidectomy effectively treats hyperparathyroidism, leading to regression of brown tumors and improvement in bone health. APTs generally have a benign course, but close follow-up is necessary due to a low (∼3%) recurrence risk, particularly in familial cases.

## Contributors

All authors made individual contributions to the work. N.S. wrote the manuscript with support from R.S., S.F., and C.S. In addition, S.F. and D.P. were involved in the diagnosis and management of the patient prior to surgery. S.R. contributed to the histopathology section and prepared the histology images. R.S. and C.S. performed the patient's surgery. All authors reviewed and approved the final draft.

## Data Availability

Original data generated and analyzed during this study are included in this published article.

## Abbreviations

APT: atypical parathyroid tumor
CT: computed tomography
HPT-JT: hyperparathyroidism-jaw tumor
PC: parathyroid carcinoma
PET-CT: positron emission tomography–computed tomography
pHPT: primary hyperparathyroidism
PTH: parathyroid hormone
